# Early post-treatment MRI predicts long-term hepatocellular carcinoma response to radiation segmentectomy

**DOI:** 10.1007/s00330-023-10045-z

**Published:** 2023-08-04

**Authors:** Daniel Stocker, Michael J. King, Maria El Homsi, Jeffrey Gnerre, Brett Marinelli, Moritz Wurnig, Myron Schwartz, Edward Kim, Bachir Taouli

**Affiliations:** 1 BioMedical Engineering and Imaging Institute, Icahn School of Medicine at Mount Sinai, New York, NY USA; 2 Institute of Diagnostic and Interventional Radiology, University Hospital Zurich, University of Zurich, Rämistrasse 100, 8091 Zurich, Switzerland; 3 Department of Diagnostic, Molecular and Interventional Radiology, Icahn School of Medicine at Mount Sinai, New York, NY USA; 4 Division of Interventional Radiology, Department of Radiology, Memorial Sloan Kettering Cancer Center, New York, NY USA; 5 Institute of Radiology, Spital Lachen AG, Lachen, Switzerland; 6 Recanati Miller Transplantation Institute, Icahn School of Medicine at Mount Sinai, New York, NY USA

**Keywords:** Liver neoplasms, Carcinoma, hepatocellular, Magnetic resonance imaging, Yttrium radioisotopes

## Abstract

**Objectives:**

Radiation segmentectomy using yttrium-90 plays an emerging role in the management of early-stage HCC. However, the value of early post-treatment MRI for response assessment is uncertain. We assessed the value of response criteria obtained early after radiation segmentectomy in predicting long-term response in patients with HCC.

**Materials and methods:**

Patients with HCC who underwent contrast-enhanced MRI before, early, and 12 months after radiation segmentectomy were included in this retrospective single-center study. Three independent radiologists reviewed images at baseline and 1^st^ follow-up after radiation segmentectomy and assessed lesion-based response according to mRECIST, LI-RADS treatment response algorithm (TRA), and image subtraction. The endpoint was response at 12 months based on consensus readout of two separate radiologists. Diagnostic accuracy for predicting complete response (CR) at 12 months based on the 1^st^ post-treatment MRI was calculated.

**Results:**

Eighty patients (M/F 60/20, mean age 67.7 years) with 80 HCCs were assessed (median size baseline, 1.8 cm [IQR, 1.4–2.9 cm]). At 12 months, 74 patients were classified as CR (92.5%), 5 as partial response (6.3%), and 1 as progressive disease (1.2%). Diagnostic accuracy for predicting CR was fair to good for all readers with excellent positive predictive value (PPV): mRECIST (range between 3 readers, accuracy: 0.763–0.825, PPV: 0.966–1), LI-RADS TRA (accuracy: 0.700–0.825, PPV: 0.983–1), and subtraction (accuracy: 0.775–0.825, PPV: 0.967–1), with no difference in accuracy between criteria (*p* range 0.053 to > 0.9).

**Conclusion:**

mRECIST, LI-RADS TRA, and subtraction obtained on early post-treatment MRI show similar performance for predicting long-term response in patients with HCC treated with radiation segmentectomy.

**Clinical relevance statement:**

Response assessment extracted from early post-treatment MRI after radiation segmentectomy predicts complete response in patients with HCC with high PPV (≥ 0.96).

**Key Points:**

• *Early post-treatment response assessment on MRI predicts response in patients with HCC treated with radiation segmentectomy with fair to good accuracy and excellent positive predictive value.*

• *There was no difference in diagnostic accuracy between mRECIST, LI-RADS, and subtraction for predicting HCC response to radiation segmentectomy.*

**Supplementary information:**

The online version contains supplementary material available at 10.1007/s00330-023-10045-z.

## Introduction

Hepatocellular carcinoma (HCC) accounts for over 80% of primary liver cancers and is estimated to be the 4th most common cause of cancer-related death worldwide [1, 2]. The arterially dominant vascular supply in HCCs makes them a favorable target for transarterial directed locoregional therapies (LRT) such as transarterial chemoembolization (TACE) or transarterial radioembolization (TARE) using yttrium-90. While TACE has established efficacy in the treatment of intermediate-stage HCC, TARE is increasingly utilized for tumor downstaging and bridging to liver transplant, in intermediate-stage HCC, as well as those with portal vein thrombosis in the advanced stage [3–5]. More recently, yttrium-90 radiation segmentectomy at the hepatic segmental level has shown an emerging role in the management of early-stage disease [6–10]. Cross-sectional imaging is not only important for pre-treatment planning but also for post-treatment response assessment, with magnetic resonance imaging (MRI) being the modality of choice in many centers.

Currently, imaging-based treatment response assessment in HCC is most commonly performed using modified Response Evaluation Criteria in Solid Tumors (mRECIST), Liver Imaging Reporting and Data System Treatment Response Algorithm (LI-RADS TRA) or by assessing the percentage of tumor necrosis using image subtraction on [11–14]. A previous study showed that response assessed early after TACE has prognostic value [15]. Due to the underlying mechanisms of action, tumor response after TARE is typically delayed [16] and the value of treatment assessment at an early time point is not well known [17, 18]. Furthermore, while the inter-reader agreement for treatment response has been evaluated in patients with HCC who underwent TACE for mRECIST [19], LI-RADS TRA [20], and subtraction [12], data for TARE is limited [21]. Although a few studies have examined the value of diffusion-weighted imaging (DWI) in predicting outcome and assessing response, its use in the clinical setting is not established [11, 22–25]. Early response prediction may help tailor individualized therapy, for example, using adjuvant therapy or early locoregional re-treatment, and may potentially improve outcome.

Therefore, the purpose of our study was to assess the value of response criteria [mRECIST, LI-RADS treatment response algorithm (TRA), and image subtraction] obtained early after radiation segmentectomy in predicting long-term response in patients with HCC.

## Materials and methods

This HIPAA-compliant, retrospective single-center study was approved by the local institutional review board with waiver of the requirement for written informed consent. The study protocol conforms to the ethical guidelines of the 1975 Declaration of Helsinki as reflected in a priori approval by the institution’s human research committee.

### Study population

**Fig. 1:**
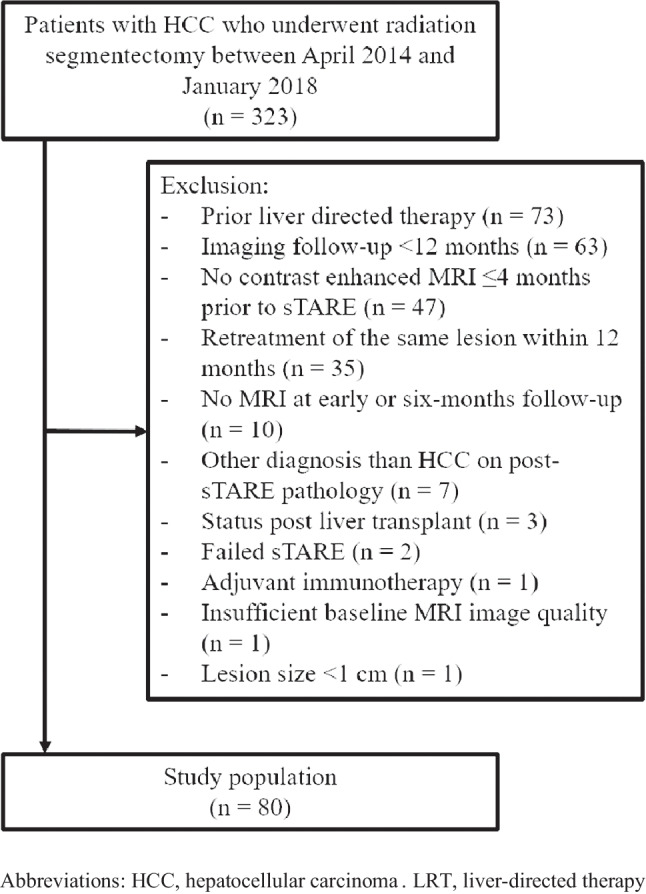
Patient flow chart

In this study, we included consecutive patients with HCC who underwent radiation segmentectomy between April 2014 and January 2018 from the interventional radiology database (*n* = 323). The diagnosis of HCC and the indication for radiation segmentectomy were based on imaging criteria (based on LI-RADS) and multidisciplinary tumor board discussion. Exclusion criteria were as follows: patients who underwent prior LRT (*n* = 73), lack of imaging follow-up at 12 months (*n* = 63), lack of contrast-enhanced MRI prior to radiation segmentectomy (*n* = 47), patients who underwent retreatment of the same lesion within 12 months (*n* = 35), lack of early post-treatment MRI (*n* = 10), other diagnosis than HCC on post-treatment pathology from liver resection (*n* = 7), status post liver transplant (*n* = 3), failed radiation segmentectomy (*n* = 2), adjuvant immunotherapy (*n* = 1), insufficient baseline MRI image quality (*n* = 1), and lesion size smaller than 1 cm (*n* = 1). The final study population consisted of 80 patients (M/F 60/20, mean age ± standard deviation 67.7 ± 9.9 years) (Fig. 1, Table 1).

**Table 1:** Characteristics of our study population

Total patients [*n*]	80
Female/male [*n*]	20 (25.0%)/60 (75.0%)
Age [years] (mean ± standard deviation)	67.7 ± 9.9 (range, 35.6–93.7)
Race/ethnicity [*n*]	
White non-Hispanic	36 (45.0%)
Hispanic	14 (17.5%)
Asian	13 (16.3%)
Black	12 (15.0%)
Other	5 (6.3%)
Underlying liver disease [*n*]	
Chronic HCV	39 (48.8%)
Chronic HBV	15 (18.8%)
NASH	12 (15.0%)
Alcohol use disorder	8 (10.0%)
Cryptogenic cirrhosis	3 (3.8%)
Other	3 (3.8%)
Child–Pugh class [*n*]	
A/B	70 (87.5%)/10 (12.5%)
BCLC stage [*n*]	
0/A/B	8 (10.0%)/61 (76.3%)/11 (13.8%)
Number of lesions [*n*]	80
Median target lesion size [cm]	1.8 (IQR 1.4–2.9)
Median AFP level at baseline [ng/mL]	9.5 (IQR 4.6–47.3)
Median injected ^90^Y dose [GBq]	1.77 (IQR 1.37–2.33)
Median delay between baseline MRI and radiation segmentectomy [days]	52.0 (IQR 39.5–62.0
Median delay between radiation segmentectomy and 1^st^ follow-up MRI [days]	47 (IQR, 43.0–53.5)
Median delay between radiation segmentectomy and 12-month follow-up [days]	393.0 (IQR 390–416)

*HCV* hepatitis C virus, *HBV* hepatitis B virus, *NASH* non-alcoholic steatohepatitis, *AFP* alpha fetoprotein, *IQR* interquartile range

### Radiation segmentectomy

Radiation segmentectomy was defined as transarterial Y90 infusion limited to two or less hepatic segments (Couinaud classification) in one treatment session [26]. The procedure and dosage methodology have been described previously [26–28]. In short, a planning angiogram was performed prior to radiation segmentectomy to identify tumor-feeding vessels and inject 99Technetium‐macroaggregated albumin (99Tc‐MAA) for shunt detection and quantification. Patients were treated with 20–30-μm-sized glass-based microspheres (TheraSphere, Boston Scientific International) to transarterially deliver Y90 to the lesion and surrounding segment. The injected activity (GBq) was dependent on lesion size and the lung shunt fraction with intended target lesion dosing > 190 Gy.

### MRI technique

The 1^st^ follow-up MRI was performed at 1.5 T (*n* = 50) or 3 T (*n* = 30) from variable vendors using gadoxetate disodium (Eovist/Primovist *n* = 69, using 10 mL fixed dose) or an extracellular contrast agent (*n* = 11, 0.1 mg/kg body weight). The median time between radiation segmentectomy and the 1^st^ follow-up MRI was 47 days (IQR, 43.0–53.5 days). Breath-hold axial pre-contrast 3D T1-weighted-images (T1WI), contrast-enhanced T1WI obtained during the arterial (2 phases back to back using fixed timing or bolus tracking method), portal venous (60 s after contrast injection), delayed/transitional phase (3 min after contrast injection) and hepatobiliary phase (only with gadoxetate disodium, 10 and 20 min after contrast injection), axial/coronal single-shot T2WI, and axial in- and opposed-phase imaging were available in all patients. Diffusion-weighted images (DWI, using b50, 400 and 800) was available in all but 6 patients. Automatically generated subtracted images (post-contrast images – pre-contrast images) were available for all contrast-enhanced phases in all patients.

### Image analysis

**Fig. 2:**
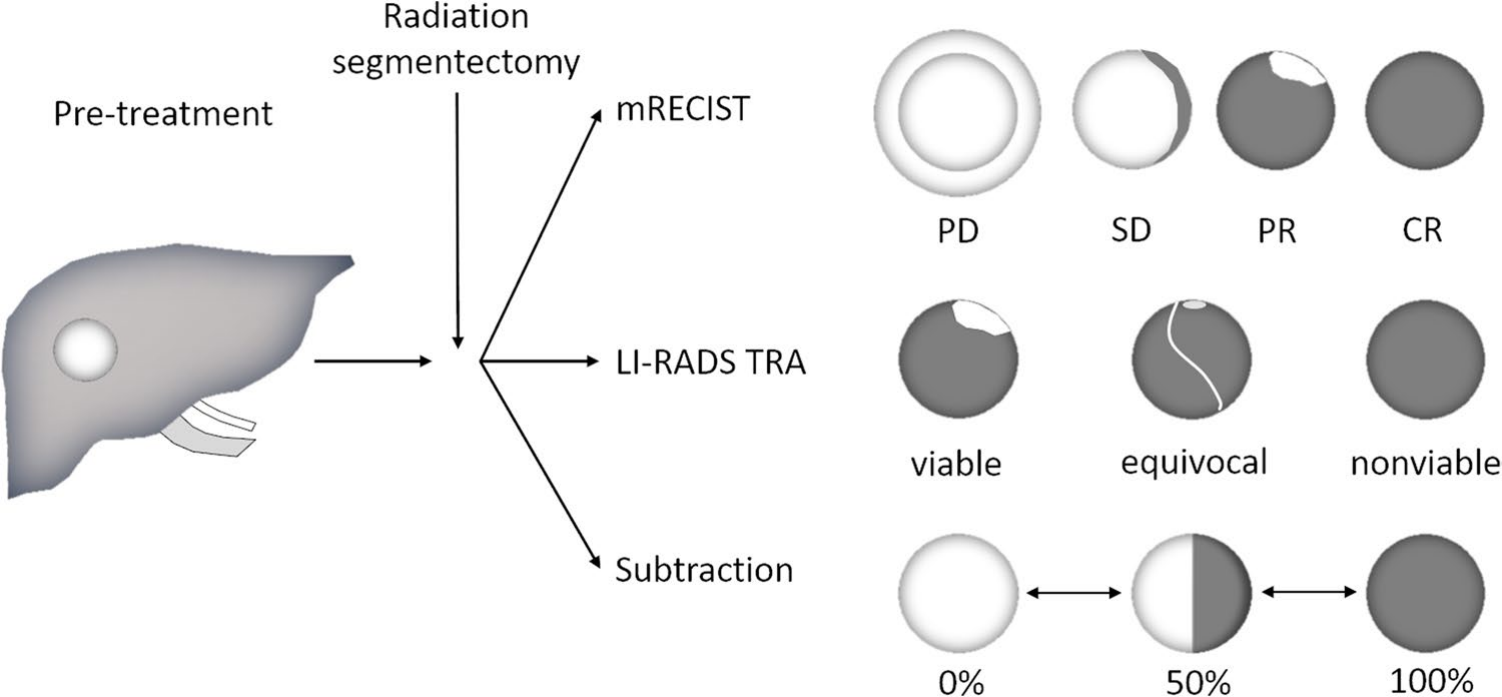
Graphic depicting the response assessment according to modified Response Evaluation Criteria in Solid Tumors (mRECIST), Liver Imaging Reporting and Data System Treatment Response Algorithm (LI-RADS TRA), and image subtraction using MRI. Note: Viable tumor is displayed in white. Necrotic tumor is displayed in dark gray

Three independent radiologists (reader 1, M.J.K., a radiologist with 4 years of post-training experience in abdominal imaging; reader 2, M.E.H., an abdominal MRI fellow with 1 year of post-training experience; reader 3, J.G., a radiologist with 2 years of post-training experience) independently reviewed all early MRI studies in random order (obtained with a median delay of 47 days post-treatment). All lesions were indicated on screen shots on baseline MRI (placed by the study coordinator, D.S.) to ensure assessment of the correct index lesions. Baseline MRI before radiation segmentectomy and the 1^st^ follow-up MRI was available for the readout. All readers were blinded to clinical data and outcome. Evaluation of response assessment for the 1^st^ follow-up was performed on mRECIST, LI-RADS TRA, and image subtraction, respectively, in a random order for each patient in the same session. The different response criteria are summarized in Fig. 1 and Supplemental Table 1 (Supplemental document). According to mRECIST, the response of each lesion was characterized as complete response (CR), partial response (PR), stable disease (SD), or progressive disease (PD), and as LR-TR nonviable, LR-TR equivocal, or LR-TR viable according to LI-RADS TRA version 2018. The percentage of necrosis was rated in 10% increments between 0 and 100% based on arterial or portal venous phase enhancement on subtraction images (Fig. 2 and Supplemental Table 1). All readers were instructed and trained using mRECIST, LI-RADS TRA, and image subtraction on a dataset (*n* = 7) that was not included in the final analysis. Tumor size was measured on portal venous phase images. We did not assess perilesional contrast enhancement in this study. Additionally, the quality of image subtraction at the 1^st^ follow-up was rated on a 5-point Likert-scale (based on liver edge visualization): 1, extensive misregistration, nondiagnostic images; 2, severe misregistration, images degraded but interpretable; 3, moderate misregistration, acceptable image quality with mild effects on diagnostic quality; 4, minimal misregistration, good image quality with minor or no effects on diagnostic quality; or 5, no misregistration, excellent image quality without effects on diagnostic quality.

### Reference standard

The reference standard was based on a consensus readout of two separate radiologists (DS, the study coordinator, and BT, with 17 years of post-training experience in abdominal imaging). Both readers were blinded to the readout results from readers 1–3. The reference standard was based on mRECIST and was categorized as CR, PR, SD, or PD, respectively. We chose mRECIST as the reference standard, because it is currently the most commonly used method to assess treatment response in HCC after LRT.

### Statistical analysis

Continuous data are reported as mean ± standard deviation or median with interquartile range (IQR) in parenthesis as appropriate. Shapiro–Wilk tests were used to test for normal distribution. Categorical data are reported as frequencies with percentages in parenthesis. Inter-reader agreement between the three readers and the agreement between the 3 different response assessment methods (mRECIST, LI-RADS TRA, subtraction) was assessed using Fleiss kappa (*κ*) [29]. A *κ* of < 0 indicated poor; 0.01–0.20, slight; 0.21–0.40, fair; 0.41–0.60, moderate; 0.61–0.80, substantial; and > 0.80, almost perfect agreement [30]. The interval data from the response assessment using image subtraction (0–100%, 10% increments) were categorized into four categories (1, 0–30%; 2, 40–60%; 3, 70–90%; 4, 100%) before calculating Fleiss kappa. To evaluate agreement between the response assessment methods and the prediction of CR at 12 months, we created binary categories (CR group and non-CR group) for the response assessment at the 1^st^ follow-up. The CR group at 1^st^ follow-up MRI was defined as CR according to mRECIST, LR-TR nonviable according to LI-RADS TRA, and 100% necrosis on subtraction, respectively. The non-CR group was defined as PR, SD, or PD according to mRECIST, LR-TR equivocal or LR-TR viable according to LI-RADS TRA, and 0–90% necrosis on subtraction, respectively. Subsequently, sensitivity, specificity, positive predictive value (PPV), negative predictive value (NPV), and accuracy with 95% confidence intervals (95% CI) were calculated for the 1^st^ follow-up MRI, using the consensus readout at 12 months as the reference standard. Differences in diagnostic accuracy between mRECIST, LI-RADS TRA, and subtraction were calculated with Cochrane *Q* tests for each reader. Statistical analysis was performed using dedicated software (IBM® SPSS® Statistics 26; SPSS® Inc.). All tests were two-tailed. A *p* value < 0.05 was considered statistically significant.

## Results

Eighty HCC lesions (right hepatic lobe, *n* = 58; left hepatic lobe *n* = 22) with a median size of 1.8 cm (IQR, 1.4–2.9 cm) were evaluated. One index lesion was evaluated per patient; in patients with more than one lesion, only the largest treated lesion was considered for treatment response evaluation. According to the consensus readout based on mRECIST at 12 months follow-up, 74 patients were classified as CR (92.5%), 5 as PR (6.3%), and 1 as PD (1.2%). No patient was classified as SD. The patient classified as PD at the 12 months follow-up showed CR on all previous follow-ups and developed local recurrence at the 12 months follow-up.

### Prediction of CR at 12 months

**Fig. 3:**
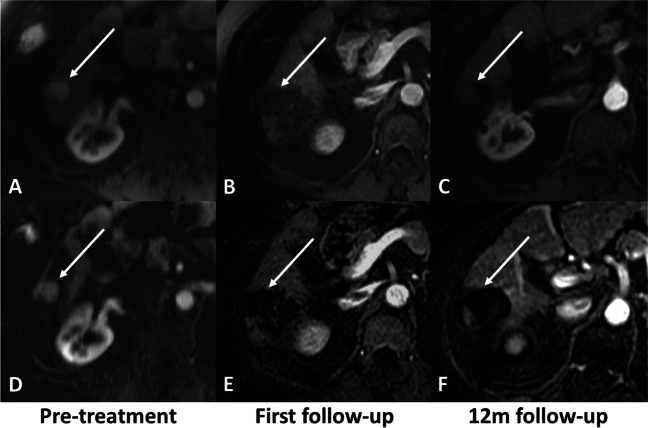
74-year-old female with NASH cirrhosis and right hepatic lobe HCC treated with radiation segmentectomy. MRI obtained during the arterial phase (upper row) and arterial phase subtraction images (lower row). Pre-treatment images show hyperenhancing lesion (arrow in **A** and **D**). 1^st^ follow-up MRI post radiation segmentectomy (**B** and **E**) demonstrates completely necrotic tumor with no residual enhancing component, and was classified as complete response according to mRECIST, LR-TR, and subtraction by the 3 readers. The treatment response at the 12-month follow-up was categorized as complete response according to mRECIST (**C** and **F**)

**Fig. 4:**
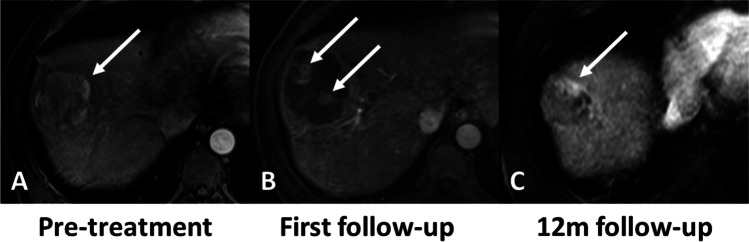
74-year-old male with NASH cirrhosis and right hepatic lobe HCC treated with radiation segmentectomy. MRI obtained during the arterial phase. Pre-treatment images show a hypervascular lesion in the right liver lobe (arrow in **A**). 1^st^ follow-up MRI post radiation segmentectomy shows two hypervascular nodules within the tumor (arrows in **B**), and was classified as partial response according to mRECIST, LR-TR equivocal (reader 1), or LR-TR viable (reader 2 and 3) according to LI-RADS TRA and 80–90% necrotic according to subtraction by the 3 readers. The treatment response at the 12-month follow-up was categorized as partial response according to mRECIST (arrow in **C**)

**Fig. 5:**
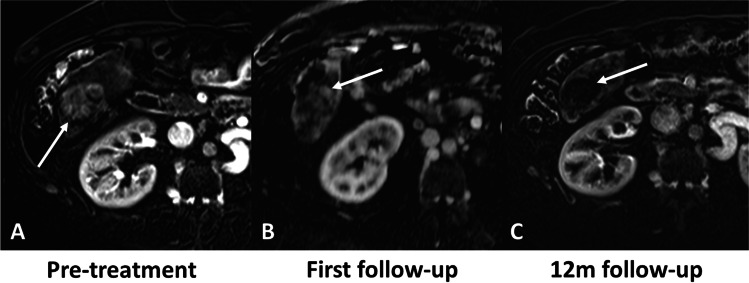
61-year-old male with HCV cirrhosis and right hepatic lobe HCC treated with radiation segmentectomy. MRI obtained during the arterial phase. Pre-treatment images show a hypervascular lesion in the right liver lobe (arrow in **A**). 1^st^ follow-up MRI show one hypervascular nodule within the tumor (arrow in **B**), and was classified as partial response according to mRECIST, LR-TR equivocal (reader 2) or LR-TR viable (readers 1 and 3) according to LI-RADS TRA and 80–90% necrotic according to subtraction by the 3 readers. The treatment response at the 12-month follow-up was categorized as complete response according to mRECIST (arrow in **C**)

Sensitivity, specificity, and PPV were fair to excellent for all readers for mRECIST (range for readers 1–3: sensitivity, 0.770–0.811; specificity, 0.667–1, and PPV, 0.966–1), LI-RADS TRA (range for readers 1–3: 0.676–0.811, 0.833–1, and 0.983–1), and subtraction (range for readers 1–3: 0.783–0.811, 0.667–1, and 0.967–1) when the 1^st^ follow-up MRI was used to predict CR at 12 months. NPV was low for all readers and methods (mRECIST, range 0.190–0.300; LI-RADS TRA, range 0.200–0.300; subtraction, range 0.200–0.300). No differences between response assessment methods regarding sensitivity, specificity, accuracy, PPV, and NPV were seen for reader 1 and reader 3. For reader 2, mRECIST and subtraction showed slightly higher sensitivity (0.770 and 0.783) and lower specificity (0.667 and 0.667) compared with LI-RADS TRA (sensitivity, 0.676; specificity, 1). No differences regarding diagnostic accuracy between mRECIST, LI-RADS TRA, and subtraction were found for reader 1 (*p* > 0.9), reader 2 (*p* = 0.053), and reader 3 (*p* > 0.9). Sensitivity, specificity, accuracy, PPV, and NPV for the prediction of CR at 12 months for all readers and criteria are presented in Table 2. An example for agreement and lack of agreement between the first follow-up and the 12-month follow-up is presented in Figs. 3, 4, and 5, respectively.

**Table 2:** Performance of response assessment criteria (mRECIST and LI-RADS TRA) and subtraction obtained from early post-treatment MRI for prediction of complete response in patients with HCC treated with radiation segmentectomy

		Sensitivity (95% CI)	Specificity (95% CI)	PPV (95% CI)	NPV (95% CI)	Accuracy (95% CI)
mRECIST	R1	0.811 (0.700, 0.890)	1 (0.517, 1)	1 (0.925, 1)	0.300 (0.128, 0.543)	0.825 (0.724, 0.901)
	R2	0.770 (0.655, 0.857)	0.667 (0.241, 0.940)	0.966 (0.872, 0.994)	0.190 (0.063, 0.426)	0.763 (0.654, 0.851)
	R3	0.797 (0.685, 0.878)	0.833 (0.365, 0.991)	0.983 (0.899, 0.999)	0.250 (0.096, 0.494)	0.800 (0.696, 0.881)
LI-RADS TRA	R1	0.811 (0.700, 0.889)	1 (0.517, 1)	1 (0.925, 1)	0.300 (0.128, 0.543)	0.825 (0.724, 0.901)
	R2	0.676 (0.556, 0.777)	1 (0.517, 1)	1 (0.911, 1)	0.200 (0.084, 0.391)	0.700 (0.587, 0.797)
	R3	0.797 (0.685, 0.878)	0.833 (0.365, 0.991)	0.983 (0.899, 0.999)	0.250 (0.096, 0.494)	0.800 (0.696, 0.881)
Subtraction	R1	0.811 (0.700, 0.889)	1 (0.517, 1)	1 (0.925, 1)	0.300 (0.128, 0.543)	0.825 (0.723, 0.901)
	R2	0.783 (0.700–0.867)	0.667 (0.241, 0.940)	0.967 (0.874, 0.994)	0.200 (0.066, 0.443)	0.775 (0.668, 0.861)
	R3	0.797 (0.685, 0.878)	0.833 (0.365, 0.991)	0.983 (0.899, 0.999)	0.250 (0.096, 0.494)	0.800 (0.696, 0.881)

Numbers in parentheses represent 95% confidence intervals. Reader’s experience: reader 1, radiologist with 4 years of post-training experience in abdominal imaging; reader 2, abdominal MRI fellow with 1 year of post-training experience; reader 3, radiologist with 2 years of post-training experience

*mRECIST* modified response criteria in solid tumors, *LIRADS TRA* liver imaging and data reporting system treatment response algorithm, *R1–3* readers 1–3, *PPV* positive predictive value, *NPV* negative predictive value

Readout results for patients with discordant response assessment between the 1^st^ follow-up and the 12-month follow-up are provided in Table 3. Patients with viable tumors at the 1^st^ follow-up but CR at the 12-month follow-up were most often rated as PR (reader 1: PR *n* = 12, SD *n* = 2; reader 2: PR *n* = 14, SD *n* = 2, PD *n* = 1; reader 3: PR *n* = 13, SD *n* = 2), viable (reader 1: viable *n* = 14; reader 2: viable *n* = 16, equivocal *n* = 8; reader 3: viable *n* = 14), and not 100% necrotic (reader 1: *n* = 14; reader 2: *n* = 16; reader 3: *n* = 15), respectively.

**Table 3:** Readout results for lesions with discordant response assessment between the 1^st^ follow-up and the 12-month follow-up. Results from the 1^st^ follow-up MRI are presented in the table

	mRECIST [*n*]	LI-RADS TR [*n*]	Subtraction [*n*]
*Lesions with viable tumor at 1*^*st*^* follow-up but CR at 12-month follow-up*			
Reader 1	PR: 12	Viable: 14	Not 100% necrotic: 14
	SD: 2	Equivocal: 0	
	PD: 0		
Reader 2	PR: 14	Viable: 16	Not 100% necrotic: 16
	SD: 2	Equivocal: 8	
	PD: 1		
Reader 3	PR: 13	Viable: 14	Not 100% necrotic: 15
	SD: 2	Equivocal: 1	
	PD: 0		
*Lesions without viable tumor at 1*^*st*^* follow-up but no CR at 12-month follow-up*			
Reader 1	CR: 0	Nonviable: 0	100% necrotic: 0
Reader 2	CR: 2	Nonviable: 0	100% necrotic: 2
Reader 3	CR: 1	Nonviable: 1	100% necrotic: 1

*mRECIST* modified response criteria in solid tumors, *LIRADS TRA* liver imaging and data reporting system treatment response algorithm, *CR* complete response, *PR* partial response, *SD* stable disease, *PD* progressive disease

### Inter-reader agreement

The inter-reader agreement between readers 1–3 was substantial for mRECIST (*κ*, 0.71; 95% CI, 0.54–0.90), LI-RADS TRA (*κ*, 0.62; 95% CI, 0.54–0.79), and subtraction (*κ*, 0.65; 95% CI 0.45–0.84). Our results suggest that experience might play a role in the response assessment with slightly higher diagnostic accuracy to predict CR for the most experienced radiologist (reader 1 with 4 years of post-training experience, 0.825), compared to the less experienced radiologist (reader 2 with 1 year of post-training experience, 0.700–0.775; reader 3 with 2 years of post-training experience, 0.800). Furthermore, the inter-reader agreement was higher for the two more experienced readers (reader 1 and 3) compared with the least experienced reader (reader 2).

### Agreement between methods

The agreement between mRECIST, LI-RADS TRA, and subtraction for the 1^st^ follow-up was almost perfect for readers 1 and 3 (both *κ*, 1.0; 95% CI 0.64–1) and substantial for reader 2 (*κ*, 0.80; 95% CI, 0.64–0.97).

### Image subtraction quality

The majority of subtracted images were rated as good or excellent quality and none as not diagnostic (Supplementary Table 2).

## Discussion

Early diagnosis of response to radiation segmentectomy is important to either avoid unnecessary retreatment or initiate additional therapy in cases without response or with suboptimal response. In our study, we found that mRECIST, LI-RADS TRA, and image subtraction assessed on early post-treatment MRI have similar performance for prediction of HCC response at 12 months after radiation segmentectomy. Furthermore, our results show that the agreement between mRECIST, LI-RADS TRA, and subtraction was substantial to almost perfect for the 3 readers. We also found that all criteria have similar substantial inter-reader agreement for the evaluation of treatment response in patients with HCC who underwent radiation segmentectomy. We also found that radiation segmentectomy is an effective treatment method with the majority of patients showing CR (92.5%) or PR (6.3%) at 12 months. These results are similar to better when compared with a previous study with a similar patient population where 84% showed CR and 4.3% showed PR after TARE and a recent study using radiation segmentectomy where 83% showed CR and 17% showed PR [6, 31].

Previous studies found that early imaging-based response assessment after LRT may have value in predicting patient outcome [15, 32, 33], which is in line with our results. In our study, sensitivity, specificity, and PPV were fair to excellent for all methods and readers when the initial post-treatment MRI was used to predict CR at 12 months. The observed high PPV (> 0.9 for all methods and readers), suggests that the majority of patients without a visible viable tumor at the initial follow-up MRI have CR at 12 months. Therefore, most patients with lesions categorized as CR, LR-TR non-viable or 100% necrotic, respectively, at early post radiation segmentectomy. MRI are still CR at 12 months (96.6–100%, depending on the reader and response assessment method). On the other hand, we found low NPV at early follow-up (< 0.3) for all methods and readers. This means that a significant number of lesions show some signs of viable tumor on early follow-up MRI after radiation segmentectomy which resolve over time and completely disappear at later time points without additional treatment. This likely reflects the delayed treatment response after TARE often showing patchy necrosis with variable residual enhancing areas [16, 34, 35]. Furthermore, in contrast to other LRTs for HCC (e.g., TACE), TARE has a minimal embolic effect and enhancing areas within a successfully treated lesion may persist on early follow-up imaging. Hence, it is of utmost importance that radiologists evaluating treatment response after TARE are aware of such expected early post-treatment changes that do not necessarily indicate residual viable tumor.

In our study, inter-reader agreement was higher compared with the current literature for mRECIST (our results: *κ*, 0.71; vs. *κ*, 0.34–0.56 [21, 36]) and LI-RADS TRA (our results: *κ*, 0.62, vs. *κ*, 0.48 [21]) in patients with HCC who underwent TARE and post-treatment MRI. However, data on treatment response assessment after TARE using MRI in patients with HCC is scarce and to the best of our knowledge, only one previous study (from our group) evaluated subtraction in this setting [37]. Our results suggest that either of these response assessment methods can be used for HCC response assessment after radiation segmentectomy, with similar substantial agreement between methods, and between readers.

Recently, machine learning and deep learning algorithms have been investigated to assess tumor response and response prediction for other LRTs such as TACE [38, 39]. To the best of our knowledge, only one study evaluated radiomics for prediction of early response after selective TARE for HCC treatment [40]. The different models in this study showed promising results with areas under the receiver operating characteristic curve ranging from 0.88 to 0.94 for prediction of response from pretreatment MRI [40]. However, validation of these initial results and further studies investigating machine learning and deep learning are needed.

The results from our study suggest that in patients with HCC treated with radiation segmentectomy, CR can be reliably diagnosed on early post-treatment MRI using mRECIST, LI-RADS TRA, or subtraction, respectively. The high PPV is promising, indicating that lesions with CR at early follow-up are likely to remain CR at later time points. On the other hand, visible viable tumor on early-post treatment MRI can resolve over time without further treatment and may subsequently result in CR. Therefore, in such cases, follow-up imaging may be prudent to avoid early initiation of unnecessary retreatment. Early diagnosis of response can improve patient management by minimizing overtreatment and hence therapy-associated side effects.

Our study has several limitations. First, the retrospective design entails possible biases, most notably a selection bias. However, we used data from consecutive patients to reduce this bias. Second, only a few lesions showed PR or PD and no lesion showed SD at the 12 month follow-up which was used as a reference standard for our study. Therefore, the interpretation of the reported specificity might be limited. Third, we included patients who underwent radiation segmentectomy and our results might not be readily applicable in patients undergoing lobar TARE, which is less used for HCC. Fourth, the diagnostic and prognostic value of DWI for response assessment was not evaluated in our study.

In conclusion, mRECIST, LI-RADS TRA, and image subtraction extracted from early MRI (around 6 weeks) post-treatment show fair to good diagnostic accuracy and excellent PPV for predicting response in patients with HCC 12 months post radiation segmentectomy.

### Supplementary Information

Below is the link to the electronic supplementary material.

Supplementary file1 (PDF 119 KB)

## Abbreviations

95% CI: 95% Confidence intervals
CR: Complete response
DWI: Diffusion-weighted imaging
HCC: Hepatocellular carcinoma
IQR: Interquartile range
LI-RADS: Liver imaging reporting and data system
LRT: Locoregional therapy
mRECIST: Modified response evaluation criteria in solid tumors
MRI: Magnetic resonance imaging
NPV: Negative predictive value
PD: Progressive disease
PPV: Positive predictive value
PR: Partial response
SD: Stable disease
T1WI: T1-weighted imaging
T2WI: T2-weighted imaging
TACE: Transarterial chemoembolization
TARE: Transarterial radioembolization
TRA: Treatment response algorithm

## Declarations

### Guarantor

The scientific guarantor of this publication is Bachir Taouli.

### Statistics and biometry

One of the authors has significant statistical expertise (Moritz Wurnig).

### Methodology

• retrospective

• diagnostic or prognostic study

• performed at one institution
