# Supplementary material for: Early post-treatment MRI predicts long-term hepatocellular carcinoma response to radiation segmentectomy
Source: Eur Radiol. 2023 Aug 4;34(1):475–84. doi: 10.1007/s00330-023-10045-z (PMC10791774; doi:10.1007/s00330-023-10045-z)
Supplement: Supplementary file 1 — Supplementary file1 (PDF 119 KB) [file 330_2023_10045_MOESM1_ESM.pdf]

Early post-treatment MRI predicts long term HCC response to radiation segmentectomy.

Supplementary material

Response assessment methods:

mRECIST: mRECIST guidelines recommend measuring only the viable tumor component on the arterial phase and comparing pre- and post-treatment measurements. Based on these measurements, the treatment response can be categorized as complete response (CR), partial response (PR), stable disease (SD) or progressive disease (PD) depending on the size difference of viable tumor between pre- and post-treatment imaging [1].

LI-RADS TRA: According to LI-RADS TRA, tumors can be categorized as LR-TR Nonviable, LR-TR Equivocal or LR-TR Viable based on imaging features, such as arterial phase hyperenhancement, washout and enhancement similar to pre-treatment enhancement [2].

Subtraction: When the percentage of lesion necrosis is used to assess treatment response, the area of tumor necrosis is evaluated on subtraction images (post-contrast - pre-contrast images) and the area of lesion necrosis is graded between 0% (no lesion necrosis) and 100% (completely necrotic lesion).

Supplementary Tables

Supplementary Table 1: Overview treatment response criteria.

**mRECIST ***		**LIRADS TRA †**		**Subtraction**	
**Category**	**Definition**	**Category**	**Definition**	**Category**	**Definition**
CR	Disappearance of any intratumoral arterial enhancement in the target lesion	LR-TR nonviable	No lesional enhancement or treatment-specific expected enhancement pattern	100%	Completely necrotic lesion
PR	At least a 30% decrease in the diameter of viable (enhancement in the arterial phase) target lesion, taking as reference the baseline of the diameter of target lesion	LR-TR equivocal	Enhancement atypical for treatment-specific expected enhancement pattern and not meeting criteria for probably or definitely viable	10-90%	Partially necrotic lesion (parts of the lesion show enhancement)
SD	Any cases that do not qualify for either partial response or progressive disease	LR-TR viable	Nodular, masslike, or thick irregular tissue in or along the treated lesion with any of the following:<br>•Arterial phase hyperenhancement<br>• Washout appearance<br>• Enhancement similar to pretreatment	0%	Viable tumor
PD	An increase of at least 20% in the diameter of viable (enhancing) target lesion, taking as reference the smallest diameters of viable (enhancing) target lesion recorded since treatment started				

* adapted from [1]

† adapted from [3]

Abbreviations: mRECIST, modified response criteria in solid tumors; LIRADS TRA, liver imaging and data reporting system treatment response algorithm; CR, complete response; PR, partial response; SD, stable disease; PD, progressive disease.

Supplementary Table 2: Quality of subtraction images on the 1st follow-up MRI for reader 1 (R1), reader 2 (R2) and reader 3 (R3).

	R1	R2	R3
**Excellent [n]**	31 (38.8%)	17 (21.3%)	39 (48.8%)
**Good [n]**	31 (38.8%)	39 (48.8%)	33 (41.3%)
**Acceptable [n]**	13 (16.3%)	21 (26.3%)	7 (8.8%)
**Degrade but interpretable [n]**	5 (6.3%)	3 (3.8%)	1 (1.3%)
**Nondiagnostic [n]**	0 (0%)	0 (0%)	0 (0%)

References

1 Lencioni R, Llovet JM (2010) Modified RECIST (mRECIST) assessment for hepatocellular carcinoma. Semin Liver Dis 30:52-60

2 Kielar A, Fowler KJ, Lewis S et al (2018) Locoregional therapies for hepatocellular carcinoma and the new LI-RADS treatment response algorithm. Abdom Radiol (NY) 43:218-230

3 Radiology ACo (2018) CT/MRI LI-RADS version 2018. Available via https://www.acr.org/Clinical-Resources/Reporting-and-Data-Systems/LI-RADS/CT-MRI-LI-RADS-v2018. Accessed 03/08/2021 2021
